# Robust High-performance Dye-sensitized Solar Cells Based on Ionic Liquid-sulfolane Composite Electrolytes

**DOI:** 10.1038/srep18158

**Published:** 2015-12-16

**Authors:** Genevieve P. S. Lau, Jean-David Décoppet, Thomas Moehl, Shaik M. Zakeeruddin, Michael Grätzel, Paul J. Dyson

**Affiliations:** 1 Institut des Sciences et Ingénierie Chimiques, Ecole Polytechnique Fédérale de Lausanne (EPFL), CH-1015 Lausanne, Switzerland

## Abstract

Novel ionic liquid-sulfolane composite electrolytes based on the 1,2,3-triazolium family of ionic liquids were developed for dye-sensitized solar cells. The best performing device exhibited a short-circuit current density of 13.4 mA cm^−2^, an open-circuit voltage of 713 mV and a fill factor of 0.65, corresponding to an overall power conversion efficiency (PCE) of 6.3%. In addition, these devices are highly stable, retaining more than 95% of the initial device PCE after 1000 hours of light- and heat-stress. These composite electrolytes show great promise for industrial application as they allow for a 14.5% improvement in PCE, compared to the solvent-free eutectic ionic liquid electrolyte system, without compromising device stability.

Harvesting energy efficiently from the sun has been a long-time goal of scientists and engineers. Among the various solar energy conversion technologies presently available, few are cost-competitive and their widespread commercial use is therefore constrained. In this regard, dye-sensitized solar cells (DSCs) are very attractive, as they are relatively low cost and easy to manufacture^1^,^2^,^3^. Furthermore, their semi-transparent and colored nature makes them ideally suited to building-integrated photovoltaic (BIPV) applications^4^.

While the best performing DSCs deliver up to 13% power conversion efficiency under full sun illumination, these high-performance devices usually employ volatile organic solvents in the electrolytes, such as acetonitrile, which greatly reduce their stability and lifetime^5^. Overcoming this limitation is therefore of paramount importance for the successful translation of this technology to the market.

Ionic liquid-based electrolytes, on the other hand, have been shown to provide DSCs with excellent long-term device stability^6^,^7^,^8^,^9^. The high ionic conductivity, wide electrochemical window, high thermal stability and non-volatile nature of ionic liquids are highly desirable for electrochemical applications, including DSCs, batteries and supercapacitors^7^,^8^,^9^,^10^,^11^. Most ionic liquids, however, are highly viscous and prevent efficient charge transport of the redox couple within the electrolyte, resulting in much lower efficiencies compared to devices based on traditional organic solvents^7^. Research in this field has been largely restricted to ionic liquids based on a limited selection of cations and anions, with the imidazolium salts being the most widely used^6^,^7^,^8^,^9^. Ionic liquids based on the 1-ethyl-3-methyl imidazolium cation have been extensively investigated in electrolytes for use in DSCs, and is frequently used as a benchmark^6^. In contrast, there are comparatively few reports on the application of low viscous 1,2,3-triazolium-based ionic liquid electrolytes in DSCs, though they have been shown to afford similarly good performance^7^. Recently, we described the synthesis of some bicyclic 1,2,3-triazolium ionic liquids and demonstrated their successful application as electrolytes in DSCs^7^. Herein, we report on a method to improve overall device performance through the addition of a plasticizer to the bicyclic 1,2,3-triazolium ionic liquid-based electrolytes, with no loss in device stability.

## Results

**Figure 1:**
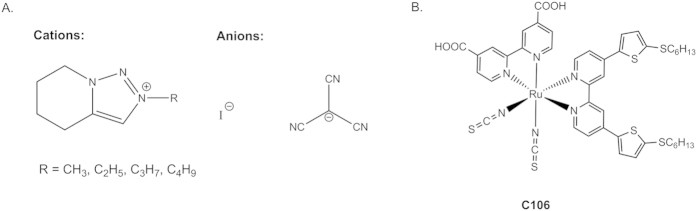
(**A**) Structures of 1,2,3-triazolium-based salts used in this study, (**B**) Structure of ruthenium dye, **C106**.

A series of electrolytes that incorporate ionic liquids based on the bicyclic 1,2,3-triazolium cations (Fig. 1A) were prepared. One set of electrolytes followed the standard composition for solvent-free eutectic ionic liquid electrolytes, coded **E1** to **E4** (see Methods for composition details). It was previously shown that a linear, inverse correlation exists between the viscosity of the electrolyte and the volume percentage of sulfolane in the electrolyte^12^. Therefore, to achieve the best photovoltaic performance, 50 vol% of sulfolane was added to electrolytes **E1** to **E4**, to obtain a second set of electrolytes (coded **ES1** to **ES4**). The triiodide diffusion coefficients in each electrolyte were determined via cyclic voltammetry and by applying equation (1), see Table 1.


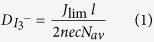


While there does not appear to be a clear trend between the triiodide diffusion coefficients and the length of the alkyl chain on the ionic liquid cation, the results show that addition of 50 vol % sulfolane to the electrolytes results in a considerable increase in the triiodide diffusion coefficient, in some cases by up to 6 times (**ES1**). A higher triiodide diffusion coefficient is typically indicative of better overall device performance due to faster transport and dye regeneration kinetics^13^.

### Photovoltaic Performance and Stability

DSCs were fabricated with a ruthenium-based photosensitizer (**C106**, Fig. 1B) using a 4 μm transparent mesoporous TiO_2_ film and electrolytes **E1–E4** and **ES1–ES4**, affording devices **A** to **H**, Table 2. Notably, device performance (power conversion efficiency, PCE) does not correlate well with the triiodide diffusion coefficients measured. However, electrolytes **E2** and **ES2** (which contain ionic liquids bearing the C2 alkyl chain) are clearly superior to the other electrolytes, giving PCEs of 5.5% and 6.3% under standard AM 1.5G illuminations at 100 mW cm^−2^, respectively. Furthermore, we note that devices which employed the sulfolane-based electrolytes (devices **E**–**H**) exhibited higher *V*_OC_ values compared to the devices which employed the solvent-free eutectic ionic liquid electrolytes (devices **A**–**D**). This difference may be ascribed to an increase in the redox energy of the electrolyte and a negative shift of the conduction band due to the use of sulfolane in the electrolyte, as described previously^12^.

The best performing electrolytes, i.e. **ES2**, **ES3** and **ES4**, were then used to fabricate DSCs with a double layer TiO_2_ film (8 + 5 μm), affording devices **I**, **J** and **K**. The application of double layer TiO_2_ films as photoanodes, in conjunction with these lower viscosity electrolytes, led to enhanced PCEs in most cases, with the best performing device delivering 6.3% and 8.4% power conversion efficiencies at 100 mW cm^−2^ and 10 mW cm^−2^, respectively (device **I**).

**Figure 2:**
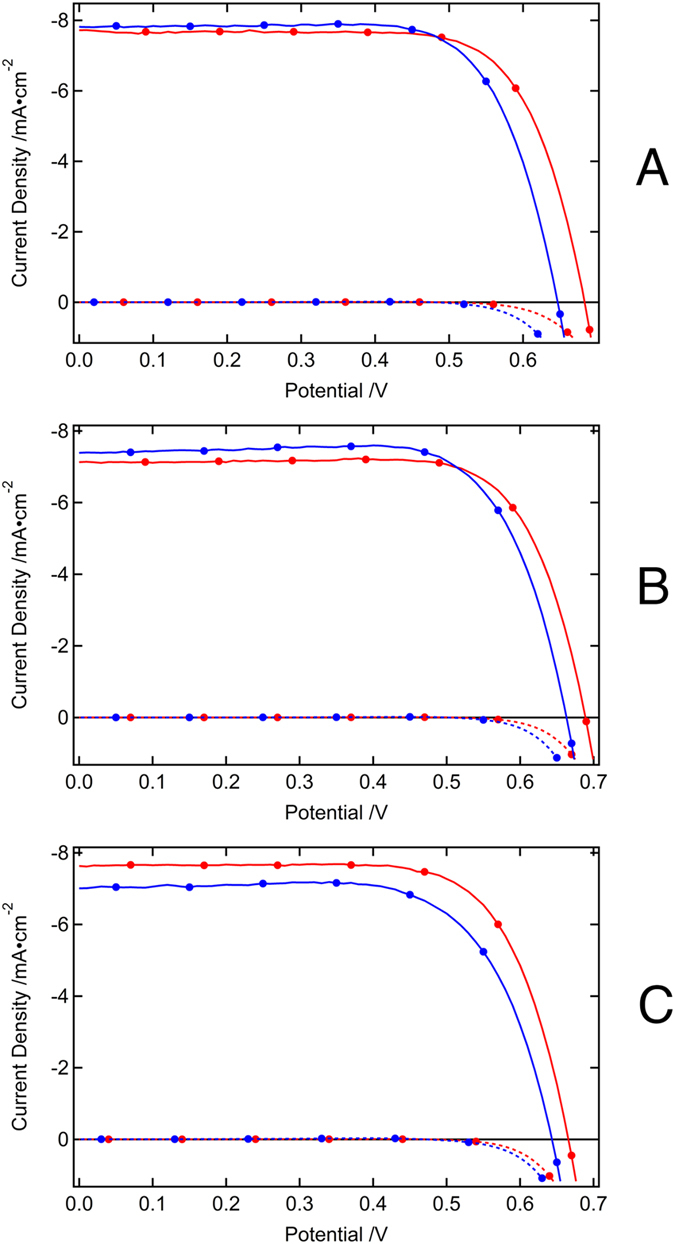
*I-V* performance of the devices (A=device I, B=device J, C=device K). Red corresponds to the initial condition and blue, the aged. The solid lines represent photocurrent, and the dotted lines represent dark current.

In order to investigate the long-term stability of these sulfolane-based DSCs, devices **I**, **J** and **K** were subjected to an accelerated aging test at 60 °C under constant full sun illumination (100 mW cm^−2^) for 1000 hours. Figure 2 shows the *I-V* performance of devices **I**, **J** and **K** before and after light-soaking, measured at 50 mW cm^−2^ illumination. The variations in the photovoltaic parameters, measured at 50 mW cm^−2^, are presented in Table 3.

**Figure 3:**
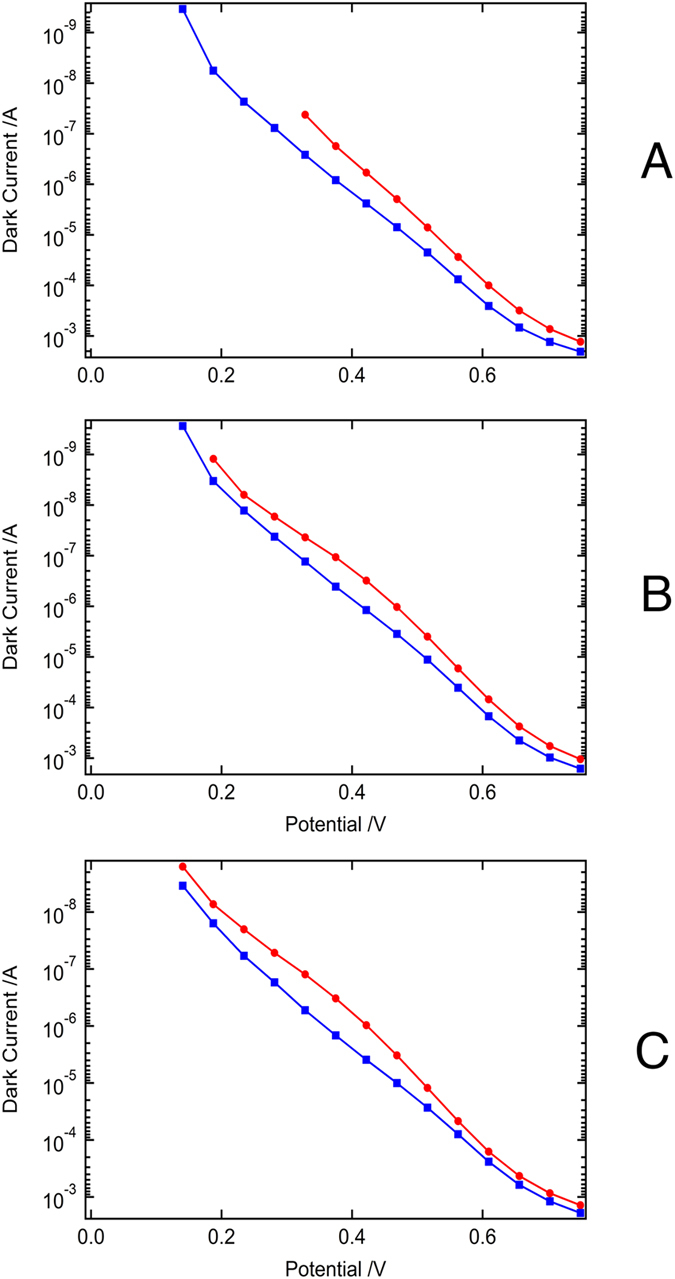
Dark current characteristics of the devices (A = device I, B = device J, C = device K). Red corresponds to the initial state and blue shows the aged dark current curve.

Although a 30 mV drop in the *V*_OC_ was observed in devices **I** and **J**, this was partially compensated for by a slight increase in the *J*_SC_ values, thus allowing for the retention of more than 95% of the initial device PCE after the 1000 hours accelerated aging test. The initial *J*_SC_ values for devices **I**, **J** and **K** were in the range of 7–8 mA cm^−2^, and the *V*_OC_ values were in the range of 660–680 mV, when measured under standard AM 1.5G illuminations at 50 mW cm^−2^. After aging the devices under full sunlight intensity at 60 °C, the changes in the *J*_SC_ values observed were minor compared to the decrease in the *V*_OC_ values. After light-soaking treatment, a decrease in *V*_OC_ of 36, 24, and 22 mV was observed for devices **I**, **J**, and **K**, respectively (Fig. 2). The drop in *V*_OC_ is in agreement with the changes observed in the dark current of the devices during the light-soaking tests (Fig. 3). In general, the aged devices show a higher dark current compared to the freshly prepared devices, presumably originating from a lower recombination resistance or from a downward shift in the TiO_2_ conduction band^14^.

Electrochemical impedance spectroscopy (EIS) studies were undertaken to help understand the changes in the photovoltaic parameters of devices **I**, **J** and **K** during the extended light-soaking treatment. EIS measurements give direct access to information regarding the charge transfer resistance of the TiO_2_/dye/electrolyte interface, the transport resistance for electrons in the mesoporous TiO_2_, and the chemical capacitance from the filling of trap states in the mesoporous metal oxide. EIS investigations were performed in the dark on devices **I**–**K**. The EI spectra were fitted according to the transmission line model^15^,^16^,^17^. The apparent electron lifetime (τ_n_) and transport time (τ_trans_) were estimated using the transport and recombination resistance, R_trans_ and R_ct_ respectively, in conjunction with the chemical capacitance (C_chem_) of the TiO_2_ (τ_n_ = R_ct_ × C_chem_ and τ_trans_ = R_trans_ × C_chem_)^15^,^16^,^17^.

**Figure 4:**
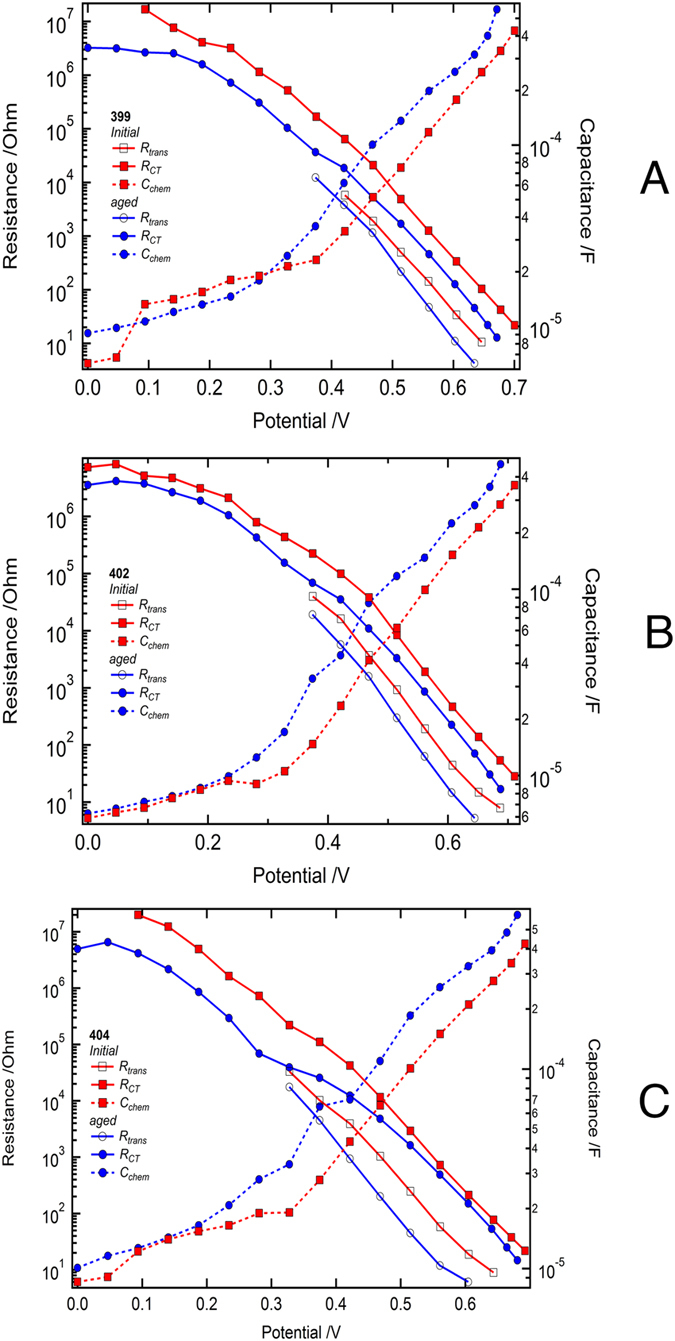
Main parameters from the EIS analysis (A = device I, B = device J, C = device K). The recombination resistance (R_ct_) clearly mirrors the observed tendencies of the dark current. Also, the change in the chemical capacitance can be observed.

The main parameters (charge transfer resistance, charge transport resistance and chemical capacitance of the mesoporous TiO_2_ film) extracted from the Nyquist plots by the transmission line model for devices **I**, **J** and **K** are presented in Fig. 4. After aging, a shift in the chemical capacitance of approximately 36, 35, and 37 mV is observed in devices **I**, **J**, and **K**, respectively (Fig. 4). These values indicate a shift in the conduction band edge of the TiO_2_ and are close to the observed differences in the *V*_OC_ before and after aging.

**Figure 5:**
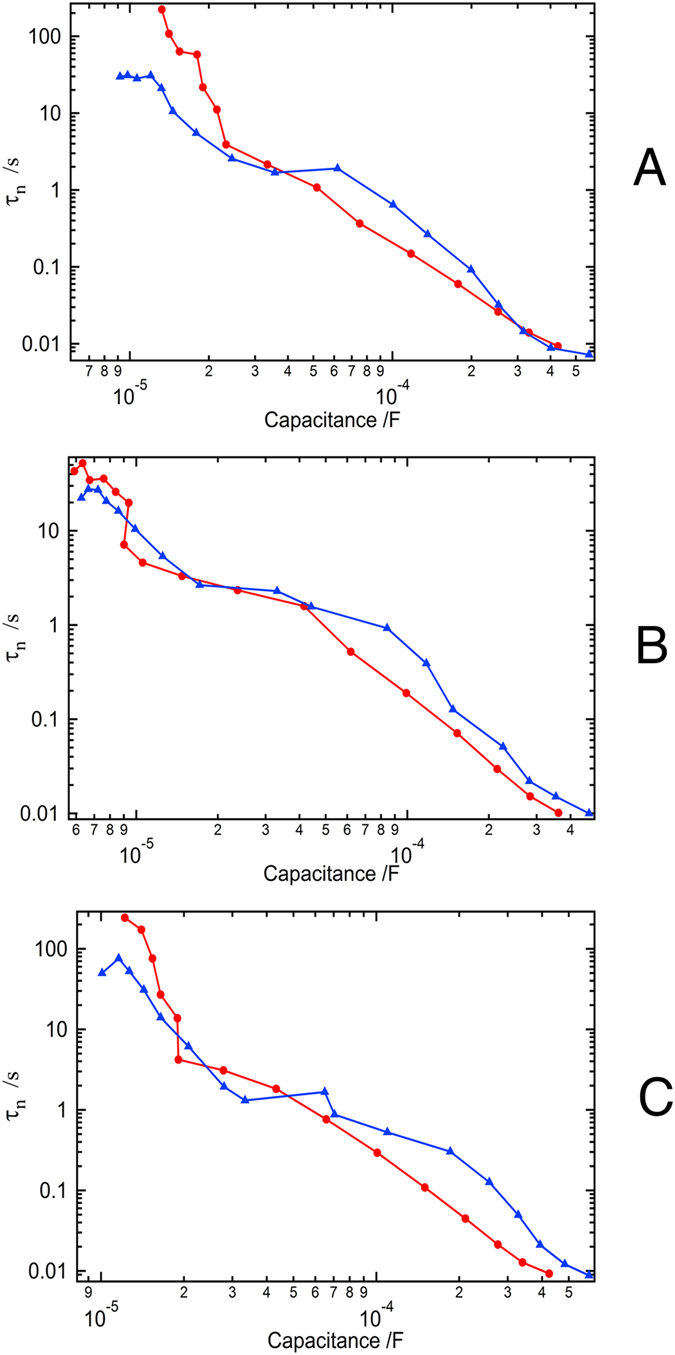
Electron lifetime of the fresh (red) and aged (blue) devices (A = device I, B = device J, C = device K).

Plotting the electron lifetime τ_n_ against the chemical capacitance (to rule out the shifts in the conduction band of different devices and compare the recombination properties at a similar charge density) the main feature that changes the *V*_OC_ is the shift of the TiO_2_ conduction band (Fig. 5). For device **I** there is not much difference in the electron lifetime during the aging process. The electron lifetime increases slightly in devices **J** and **K** when aged, further reducing the loss of voltage due to a downward shift of the conduction band. Thus, the main change in *V*_OC_ originates from the conduction band shift, which is partly compensated for in devices **J** and **K** by the increase in electron lifetime. While the length of the alkyl chain on the triazolium cation does not appear to significantly influence the position of the conduction band, a longer chain length does seem to favor longer electron lifetimes.

## Discussion

The triiodide diffusion coefficients of each electrolyte, as determined by cyclic voltammetry, confirm our hypothesis that addition of 50 vol % sulfolane to solvent-free eutectic ionic liquid electrolytes results in the lowering of their overall viscosity, in some cases by up to 6 times. This is desirable for efficient electron transfer kinetics within the device, and contributes to higher device PCEs, which were also observed experimentally. Device **B**, which contained electrolyte **E2**, gave a power conversion efficiency of 5.5% at full sun, while device **F**, which contained electrolyte **ES2**, gave a power conversion efficiency of 6.3% at full sun. This represents a 14.5% improvement when going from the solvent-free eutectic ionic liquid electrolyte system to the ionic liquid-sulfolane composite electrolyte system. Dilution of the ionic liquid-based electrolytes with sulfolane is therefore a simple and straightforward method to reduce overall device costs while improving device performance.

Long-term stability tests performed on DSCs containing the novel triazolium ionic liquid-sulfolane composite electrolytes showed that the sulfolane-based systems are in fact very robust, retaining more than 95% of their initial PCE after 1000 hours of continuous light- and heat-stress (devices **I** and **J**).

In conclusion, we have developed novel triazolium ionic liquid-sulfolane composite electrolytes for application in dye-sensitized solar cells. Devices employing these new electrolytes exhibit very good power conversion efficiencies and device stability, comparable to existing benchmarks.

## Methods

### Reagents and materials

1,3-dimethylimidazolium iodide (DMII) was purchased from Merck. The **C106** dye, dineohexyl bis-(3,3-dimethyl-butyl)-phosphinic acid (DINHOP), and the bicyclic triazolium ionic liquids used were prepared as reported earlier^7^,^18^,^19^. Sulfolane (99%) was purchased from Aldrich and distilled before use. All other reagents were obtained from commercial sources and used as received.

### Photovoltaic device fabrication

Dye-sensitized solar cells were fabricated using a double-layered photoanode made of mesoporous TiO_2_ as reported earlier^7^. Prior to use the photoanodes were briefly sintered again and after cooling to 80 °C immersed for 16 h at room temperature in a 0.3 mM **C106** dye solution in 10% DMSO and tert-butanol:acetonitrile (1 :1 v/v) with DINHOP as a co-adsorbent, the molar ratio of dye to DINHOP being 4:1. The dye-loaded substrates were then rinsed with acetonitrile, dried and subsequently sealed with pieces of thermally platinized (a drop of 8 mM hexachloroplatinic solution in 2-propanol, heated to 425 °C) FTO glass (TEC15, Pilkington), which served as a counter electrode. 25-μm-thick Surlyn (Dupont) was used as a binder and a spacer. The electrolytes were introduced into the cells via pre-drilled holes in the counter electrodes. The ionic liquid-based electrolytes had the following general composition: DMII/GNCS/NMB/**IL-1**/**IL-2**/I_2_ (6 : 0.33 : 1.74 : 6:8 : 1.2 by mol, DMII = 1,3-dimethylimidazolium iodide, GNCS = guanidinium thiocyanate, NMB = N-methylbenzimidazole, **IL-1** = BT-C1^+^I^–^, **IL-2** = BT-C1^+^TCM^–^). Four different electrolytes were prepared, differing in the length of the alkyl chain on the cations in **IL-1** and **IL-2**, from C1 to C4, giving electrolytes **E1–E4.** To obtain the sulfolane-based electrolytes, **ES1–ES4**, 50 volume % of sulfolane was added to **E1–E4**, respectively.

### Photovoltaic measurements and accelerated aging tests

An AM 1.5 solar simulator equipped with a 450 W Xenon lamp (Oriel, USA) was used for all photovoltaic measurements. To obtain the *I–V* curves, an external bias was applied to the cell and the generated photocurrent was measured with a Keithley model 2400 digital source meter. All devices were masked to attain an illuminated active area of 0.159 cm^2^. For the accelerated aging tests, the devices were placed under continuous full sun illumination (100 mW cm^−2^) at 60 °C, in the presence of a UV cut-off filter. The devices were kept under open circuit conditions throughout the experiment, and were periodically removed for measurements.

### Electrochemical impedance spectroscopy (EIS) measurements

EIS measurements were performed with a BioLogic SP300 potentiostat providing a voltage modulation of 15 mV in the desired frequency range (1 MHz to 0.1 Hz). The EIS measurements of the devices made for the determination of the diffusion coefficient were performed at 0 V. Z-view software (v2.8b, Scribner Associates Inc.) was used to analyze the impedance data on the basis of Randles circuit. The impedance spectra of the measured devices were recorded at bias potentials varying from 0 to 750 mV in 50 mV steps. The impedance spectra of the DSCs were analyzed on the basis of the transmission line model^15^,^16^,^17^.

## Additional Information

**How to cite this article**: Lau, G. P. S. *et al.* Robust High-performance Dye-sensitized Solar Cells Based on Ionic Liquid-sulfolane Composite Electrolytes. *Sci. Rep.* **5**, 18158; doi: 10.1038/srep18158 (2015).

## Figures and Tables

**Table 1:** The electrolytes triiodide diffusion coefficient determined by cyclic voltammetry.

Electrolyte*	Triiodide Diffusion Coefficient [cm^2^ s^–1^]
E1	5.67e-08
E2	9.46e-08
E3	5.83e-08
E4	6.78e-08
ES1	3.78e-07
ES2	3.26e-07
ES3	1.36e-07
ES4	3.59e-07

^*^E1,**E2**,**E3** and **E4** contain 0.33 M I_2_ and **ES1**, **ES2**, **ES3** and **ES4** contain 0.165 M I_2_.

**Table 2:** Photovoltaic parameters of devices A–H measured under standard AM 1.5G illuminations at 100 mW cm^–2^ and 50 mW cm^–2^.

Device	Alkyl chain	Electrolyte	Light intensity [mW cm^–2^]	J_sc_[mA cm^–2^]	V_oc_ [mV]	Fill Factor	PCE [%]
**A**	C1	**E1**	100	8.6	680	0.76	4.7
			50	5.5	667	0.72	5.3
**B**	C2	**E2**	100	10.7	696	0.71	5.5
			50	5.7	679	0.76	5.9
**C**	C3	**E3**	100	5.9	690	0.87	3.6
			50	4.8	674	0.81	5.2
**D**	C4	**E4**	100	6.4	675	0.83	3.7
			50	5.2	668	0.74	5.2
**E**	C1	**ES1**	100	12.3	709	0.62	5.5
			50	6.8	694	0.70	6.5
**F**	C2	**ES2**	100	13.4	713	0.65	6.3
			50	7.1	695	0.74	7.1
**G**	C3	**ES3**	100	11.4	733	0.69	5.9
			50	7.0	717	0.72	7.1
**H**	C4	**ES4**	100	13.3	715	0.64	6.2
			50	7.2	696	0.72	7.1

**Table 3:** Photovoltaic parameters of devices I, J and K measured at 50 mW cm^-2^, before and after aging under standard AM 1.5G illuminations at 100 mW cm^-2^ and 60 °C.

Device	Alkyl chain	Electrolyte	Aging [h]	*J*_SC_ [mA cm^–2^]	*V*_OC_ [mV]	Fill Factor	PCE [%]	E_cb_ shift	Δ(E_cb_ Shift–*V*_OC_)
I	C2	ES2	0	7.82	682	0.73	7.87	–	0
			1000	7.91	646	0.72	7.50	36	
J	C3	ES3	0	7.22	688	0.74	7.49	–	10
			1000	7.48	663	0.73	7.34	35	
K	C4	ES4	0	7.73	665	0.72	7.50	–	15
			1000	7.10	643	0.70	6.45	37	
